# Epithelial HO-1 regulates iron availability and promotes colonic tumorigenesis in a context-dependent manner

**DOI:** 10.1172/jci.insight.181032

**Published:** 2025-12-17

**Authors:** Rosemary C. Callahan, Jillian C. Curry, Geetha Bhagavatula, Alyse W. Staley, Rachel E.M. Schaefer, Faiz Minhajuddin, Liheng Zhou, Rane M. Neuhart, Shaikh M. Atif, David J. Orlicky, Ian M. Cartwright, Mark E. Gerich, Calen A. Steiner, Arianne L. Theiss, Caroline H.T. Hall, Sean P. Colgan, Joseph C. Onyiah

**Affiliations:** 1 Division of Gastroenterology and Hepatology, Department of Medicine, University of Colorado School of Medicine, Aurora, Colorado, USA.; 2 Rocky Mountain Regional Veterans Affairs Medical Center, Aurora, Colorado, USA.; 3 Division of Gastroenterology, Hepatology and Nutrition, Department of Pediatrics, Children’s Hospital Colorado; Mucosal Inflammation Program, Department of Medicine, University of Colorado School of Medicine, Aurora, Colorado, USA.; 4 University of Colorado Cancer Center Biostatistics Core, Aurora, Colorado, USA.; 5 Division of Allergy and Clinical Immunology, Department of Medicine, and; 6 Department of Pathology, University of Colorado School of Medicine, Aurora, Colorado, USA.

**Keywords:** Cell biology, Gastroenterology, Cell stress, Colorectal cancer, Inflammatory bowel disease

## Abstract

Induction of heme oxygenase-1 (HO-1/*Hmox1*) is broadly considered cytoprotective, but the role of colonic epithelial HO-1 in colitis-associated tumorigenesis is poorly defined. HO-1 catabolizes heme, releasing ferrous iron, a key driver of oxidative stress and lipid peroxidation. We observed that colonic epithelial HO-1 was induced during colitis and tumorigenesis. We also found that HO-1 was upregulated in ferroptosis-inducing conditions in murine and human colonic epithelial organoids and correlated with lipid peroxidation and ferroptosis markers in colonic tumors. In colonic epithelial organoids exposed to heme, deletion of *Hmox1* amplified a compensatory oxidative stress and detoxification transcriptional program, likely reflecting unresolved oxidative and nonoxidative toxicity from heme. In vivo, epithelial HO-1–deficient mice developed significantly fewer and smaller tumors compared with littermate controls in a colitis-associated tumorigenesis model, despite similar inflammatory injury. Tumors from KO mice exhibited reduced iron levels, decreased lipid peroxidation, lower oxidative DNA damage, and decreased proliferation. Single-cell RNA sequencing of tumor epithelial cells revealed a shift from a proliferative to a stress-adaptive program with loss of HO-1. These findings identify epithelial HO-1 as a context-dependent regulator of tumorigenesis: it is protective against acute heme toxicity but promotes iron-dependent oxidative damage and proliferation in the setting of chronic inflammation.

## Abstract 2

Colonic tumorigenesis in mice is influenced by epithelial heme oxygenase-1, which regulates the balance between epithelial heme metabolism and ferroptosis.

## Introduction

Inflammatory bowel disease is characterized by chronic, relapsing-remitting mucosal inflammation that drives cycles of epithelial injury and repair, significantly increasing the risk of dysplasia and colorectal cancer (1, 2). This risk is particularly elevated in patients with ulcerative colitis (UC), where disease activity, extent, and duration correlate with neoplastic transformation (3, 4). A hallmark of active colitis is mucosal hemorrhage, which exposes the intestinal epithelium to elevated levels of luminal heme released from damaged extravascular erythrocytes (5, 6). Heme is a potent prooxidant molecule capable of generating ROS via its redox active iron atom, leading to oxidative DNA damage, lipid peroxidation, and cell death (7–10).

To mitigate heme toxicity, tissues rely on binding proteins and enzymatic degradation pathways (11). Heme oxygenase-1 (HO-1/*Hmox1*) is a heme and stress-inducible enzyme that catabolizes heme into biliverdin, carbon monoxide, and ferrous iron (12). Systemic deficiency of HO-1 in humans and mice results in increased oxidative stress, inflammation, and tissue injury (13–15). While HO-1 is broadly considered cytoprotective and antiinflammatory, its role in cancer is complex and context dependent (12). In the acutely inflamed colon, HO-1 is upregulated in response to inflammation and hemorrhage, but the consequences of its chronic activation, particularly in epithelial cells, have not been well defined. In particular, the intersection of HO-1 activity with iron-regulated oxidative stress and transcriptional responses in the context of colitis-associated cancer (CAC) remains unexplored.

Iron-dependent lipid peroxidation and oxidative stress have been increasingly implicated in both inflammatory and neoplastic processes in the gut, with ferroptosis representing one potential outcome of dysregulated iron metabolism (16–19). Iron metabolism is increasingly recognized as a critical determinant of tumor behavior, with labile iron shown to support nucleotide synthesis, metabolic reprogramming, and proliferation (20, 21). Catabolism of heme by HO-1, with the release of ferrous iron, may therefore have an unintended consequence: fueling tumor-promoting pathways during chronic inflammation. Conversely, loss of HO-1 may exacerbate acute heme toxicity through oxidative and nonoxidative damage from uncatabolized heme, independent of iron release (22). This paradox underscores the need to understand the influence of epithelial HO-1 on colitis and tumorigenesis.

To address this, we utilized a murine model of CAC and generated intestinal epithelial cell–specific HO-1–KO mice (*Hmox1*^ΔIEC^ mice). We examined the effect of HO-1 deletion on tumor burden, oxidative stress, and compensatory transcriptional responses using epithelial organoids, tissue-level assays, and single-cell transcriptomics. Our findings reveal a complex role for epithelial HO-1 in colitis-associated tumorigenesis: while it protects against acute heme stress, its activity may promote oxidative damage and epithelial proliferation in chronic inflammation through increased iron availability. In its absence, heme triggers a compensatory stress-adaptive transcriptional program that constrains tumor growth.

## Results

### Loss of colonic epithelial HO-1 amplifies stress-linked transcriptional responses to heme.

**Figure 1:**
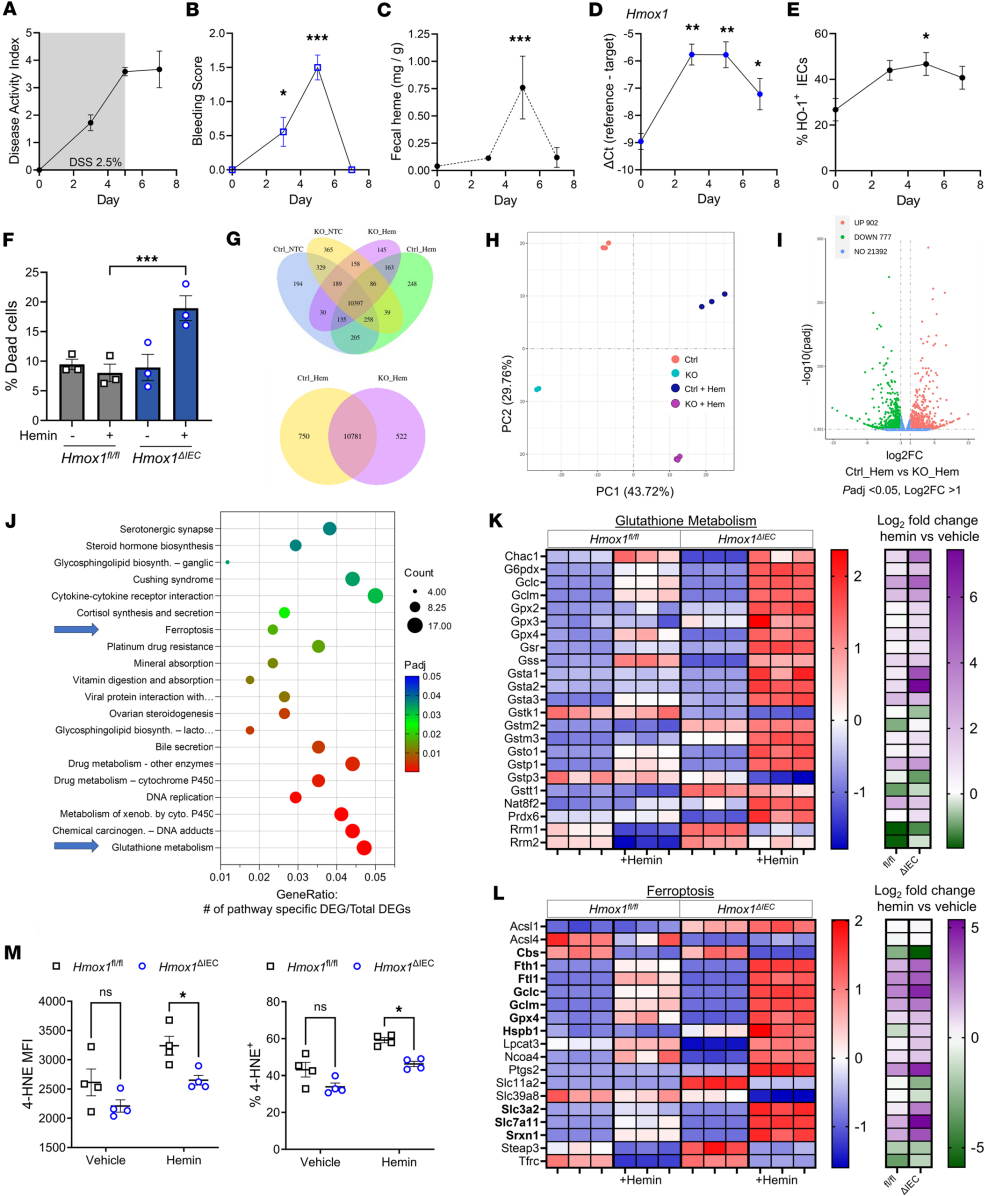
Influence of HO-1 on heme-linked stress responses in colonic epithelial cells. WT mice were given DSS 2.5% in their drinking water for 5 days with daily monitoring up to 2 days after DSS was removed (*n* = 3–9 per time point). (**A**) Disease activity index (DAI; weight, stool blood, and diarrhea). (**B**) Bleeding component of DAI. (**C**) Fecal heme quantification during DSS injury (*n* = 4–12 per group). (**D**) Whole colon mRNA expression of *Hmox1* by RT-qPCR (*n* = 3–10 per group). (**E**) HO-1 detection by flow cytometry, as a percentage of total IECs (*n* = 3 per group). (**F**) Colonic epithelial organoids (colonoids) were derived from healthy *Hmox1*^fl/fl^ and *Hmox1*^ΔIEC^ epithelial stem cells. After exposure to hemin (200 μM) for 24 hours, colonoid cell death was assessed by flow cytometry (7-AAD). (**G**) Venn diagrams of differentially expressed gene (DEG) patterns in KO and control colonoids in response to hemin, as measured by RNA sequencing. (**H**) Principal component analysis 2D plot of colonoid groups. (**I**) Volcano plot of differentially expressed genes (up, increased expression in control colonoids; down, decreased expression in control colonoids; no, no change in expression). Values show the number of DEGs in each group. (**J**) KEGG enrichment analysis showing the top 20 significant upregulated pathways in the KO colonoids treated with hemin (Benjamini and Hochberg FDR correction). Blue arrows highlight key related pathways, examined in more detail in **K** and **L**. (**K** and **L**) *Z*-score of DEGs from RNA seq of control and KO colonoids. Log_2_ fold change of comparison between vehicle- and hemin-treated groups by cell type. (**L**) Bolded genes are typically protective against ferroptosis. (**M**) Colonoids were exposed to 100 μM hemin for 24 hours, and median fluorescence intensity (MFI) and the percentage 4-HNE^+^ cells were determined using flow cytometry. Data are shown as the mean ± SEM. *P < 0.05, **P < 0.01, and ***P < 0.001, by unpaired, Student’s *t* tests (**M**) or 1-way ANOVA for multiple comparisons (**B**–**F**) (e.g., compared with day 0 or normal colon).

We examined mucosal bleeding as a feature of active colitis using the murine dextran sodium sulfate–induced (DSS-induced) colitis model. We observed a temporal increase in disease activity, fecal bleeding, and fecal heme content, which declined after DSS withdrawal (Figure 1, A–C) (23). Induction of the heme-inducible HO-1/CO pathway has been shown to be protective in murine colitis (24–26). Colonic *Hmox1* mRNA and HO-1 in CD45^–^EPCAM^+^ IECs increased during the course of DSS injury, correlating with bleeding severity and fecal heme (Figure 1, D and E). To investigate colonic epithelial responses to heme we generated mice with IEC-specific deletion of *Hmox1* expression (*Hmox1*^ΔIEC^ or KO mice; Supplemental Figure 1, A and B; supplemental material available online with this article; https://doi.org/10.1172/jci.insight.181032DS1) and derived colonic epithelial stem cell organoids (colonoids) from control (*Hmox1*^fl/fl^) and KO animals (Supplemental Figure 1C). Colonic epithelial cells are relatively resistant to hemin-induced cytotoxicity, but, upon exposure to moderate amounts of heme, we found that loss of *Hmox1* led to increased cell death in murine colonoids (Figure 1F), similar to a prior report using human colonic epithelial cell lines (8). RNA sequencing revealed distinct transcriptional responses to heme in KO versus control colonoids, with approximately 800 genes upregulated in KO and approximately 900 in controls (Figure 1, G–I). Kyoto Encyclopedia of Genes and Genomes (27) pathway analysis demonstrated enrichment of glutathione metabolism, ferroptosis-response, and several detoxification pathways in KO colonoids (Figure 1, J–L), consistent with increased activation of a broad stress response. Similar to glutathione metabolism, many of the upregulated ferroptosis-response genes are involved in the protective response against iron-induced oxidative damage, such as *Fth1* and *Ftl1*, which store ferrous iron inert as ferritin, or *Slc7a11* and *Slc3a2* that together encode the cystine transporter that is key to maintaining antioxidative glutathione levels.

qPCR analysis confirmed upregulation of antioxidant and ferroptosis-response genes in KO colonoids (Supplemental Figure 1D). Depletion of glutathione and inhibition of HO-1 triggers cell cycle arrest in epithelial cells (8, 28, 29). We performed cell cycle analysis and observed increased G_0_/G_1_ arrest in KO colonoids exposed to heme (Supplemental Figure 1E). These findings suggest that HO-1 protects colonic epithelial cells from acute heme-induced stress through heme catabolism; in its absence, persistent heme triggers a compensatory stress-adaptive transcriptional program and cell cycle arrest. To further assess iron-induced oxidative damage in response to heme, we performed flow cytometry for the lipid peroxidation byproduct 4-hydroxynonenal (4-HNE) in control and KO colonoids exposed to hemin. KO colonoids exhibited significantly reduced 4-HNE levels (median fluorescence intensity) and a reduced percentage of positive cells compared with controls (Figure 1M), suggesting that heme-iron released by HO-1 contributes to lipid peroxidation in colonic epithelial cells. These results reinforce the role of HO-1 in regulating oxidative stress responses and susceptibility to lipid damage. However, increased cell death in the KO colonoids points to nonoxidative injury, likely induced by ongoing heme stress.

### HO-1 expression is induced alongside glutathione and ferroptosis response pathways in murine and human colonic tissue and organoids.

**Figure 2:**
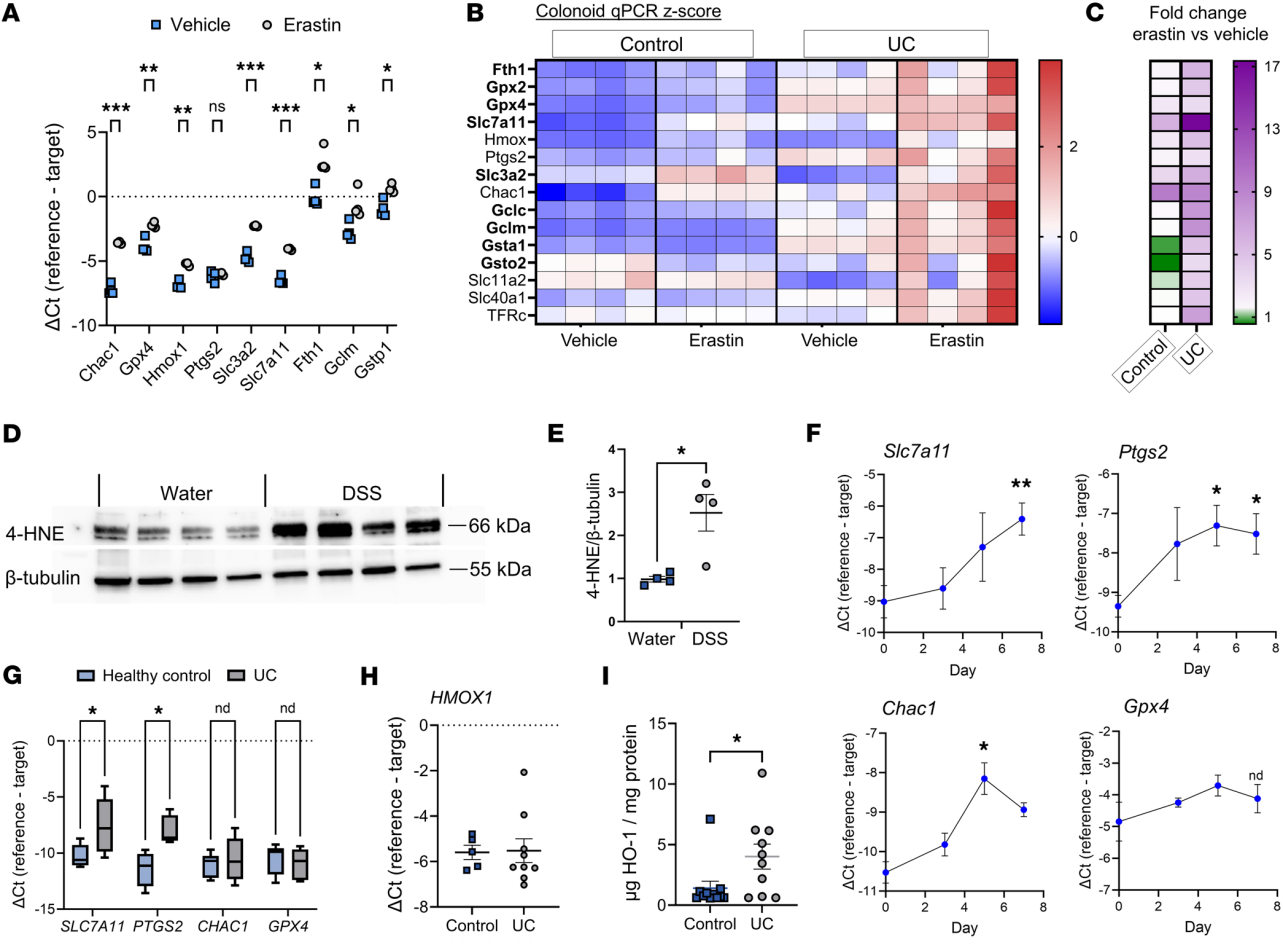
HO-1 is induced alongside genes associated with ferroptosis and antioxidant responses in murine and human colonic tissue and organoids. (**A**) Murine colonoid mRNA expression by RT-qPCR after exposure to vehicle or erastin (20 μM) for 24 hours. (**B**) *Z*-score of gene expression in healthy human vs. UC-derived colonoid by RT-qPCR after exposure to vehicle or erastin (20 μM) for 24 hours Genes associated with ferroptosis and antioxidant responses are shown in bold. (**C**) Log_2_ fold change reflects comparison between erastin- and vehicle-treated groups within each cell type only. (**D** and **E**) WT mice were given DSS 2.5% in their drinking water for 5 days, and colonic tissue was collected and analyzed by Western blot for ferroptosis marker 4-HNE compared with mice only given drinking water alone. Levels of 4-HNE were calculated relative to β-tubulin (*n* = 4). (**F**) Whole colon mRNA expression by RT-qPCR of tissue from WT mice that were given DSS 2.5% in their drinking water for 5 days followed by drinking water alone for 2 days after DSS was removed (*n* = 3–4 mice per time point). (**G**) Human colonic biopsy tissue mRNA expression by RT-qPCR (*n* = 4–6 control, 7–9 UC). (**H**) *HMOX*1 mRNA from human colonic biopsy tissue. (**I**) Human colonic biopsy tissue HO-1 assessed by ELISA of tissue homogenates relative to total protein. Data are shown as the mean ± SEM. *P < 0.05, **P < 0.01, and ****P < 0.0001 by unpaired Student’s *t* test (**A**, **E** and **G**–**I**) and multiple *t* test for 2 groups and ANOVA (**F**) for 3 or more groups with correction for multiple comparisons.

Given the activation of glutathione metabolism and ferroptosis-response pathways in HO-1–deficient colonoids in response to heme (Figure 1), we examined whether similar stress responses are activated in colonic epithelial cells in response to induction of ferroptosis and inflammation. WT murine colonoids were exposed to erastin, a glutathione-depleting compound that induces oxidative stress and eventually ferroptosis by inhibiting the cystine/glutamate antiporter system X_C_^–^ encoded by *Slc7a11* and *Slc3a2* (16). There was a significant upregulation of ferroptosis-response and glutathione metabolism genes, along with increased *Hmox1* expression (Figure 2A). Similarly, human colonoids derived from biopsies from patients with UC exhibited higher baseline and erastin-induced expression of ferroptosis and glutathione metabolism genes compared with colonoids from healthy control biopsies (Figure 2, B and C). This includes *HMOX1*, suggesting that HO-1 is consistently upregulated in epithelial cells under proferroptotic conditions.

Lipid peroxidation has recently been identified as a feature of intestinal inflammation (17). We assessed the physiological relationship of HO-1 induction seen in DSS colitis (Figure 1) to markers of ROS-mediated lipid injury in vivo. During the course of DSS injury, we observed increased levels of 4-HNE (Figure 2, D and E), along with increased expression of well-reported marker genes, including *Ptgs2*, *Slc7a11*, and *Chac1* (Figure 2F) (18). Gene expression in UC biopsy tissue showed similar trends, with increased ferroptosis responsive genes such as *PTGS2* and *SLC7A11* (Figure 2G). We examined a publicly available Gene Expression Omnibus data set (GSE38713) that reported transcriptional patterns in colon tissue from healthy individuals and from patients with UC (30). A similar elevation of ferroptosis-linked gene expression was seen, particularly in actively inflamed tissue from patients with UC, similar to changes seen at the peak of DSS injury on day 7 (Supplemental Figure 2). In our own tissue samples from healthy individuals and those with UC, we detected increased expression of HO-1 protein consistent with prior studies in patients with UC (31–33), while *HMOX1* mRNA remained unchanged suggestive of posttranscriptional regulation (Figure 2, H and I). Together, these results demonstrate that HO-1 induction correlates with activation of glutathione metabolism pathways and intersects with the ferroptosis-response pathway in the context of experimental and disease-associated inflammation and oxidative stress.

### HO-1 and ferroptosis markers are upregulated in colonic tumors and correlate with lipid peroxidation.

Chronic colitis is a well-established risk factor for colorectal cancer, primarily due to persistent oxidative stress and cycles of epithelial injury and repair. Emerging evidence implicates lipid peroxidation in the pathogenesis of tumorigenesis (34). Developing tumors often exhibit enhanced resistance to oxidative stress, facilitated by upregulation of antioxidative pathways that support cancer stem cell survival and proliferation (35). Paradoxically, colorectal cancer cells also exhibit a heightened demand for iron to sustain metabolic activity and growth, despite the risk of iron-induced oxidative damage (20, 36).

**Figure 3:**
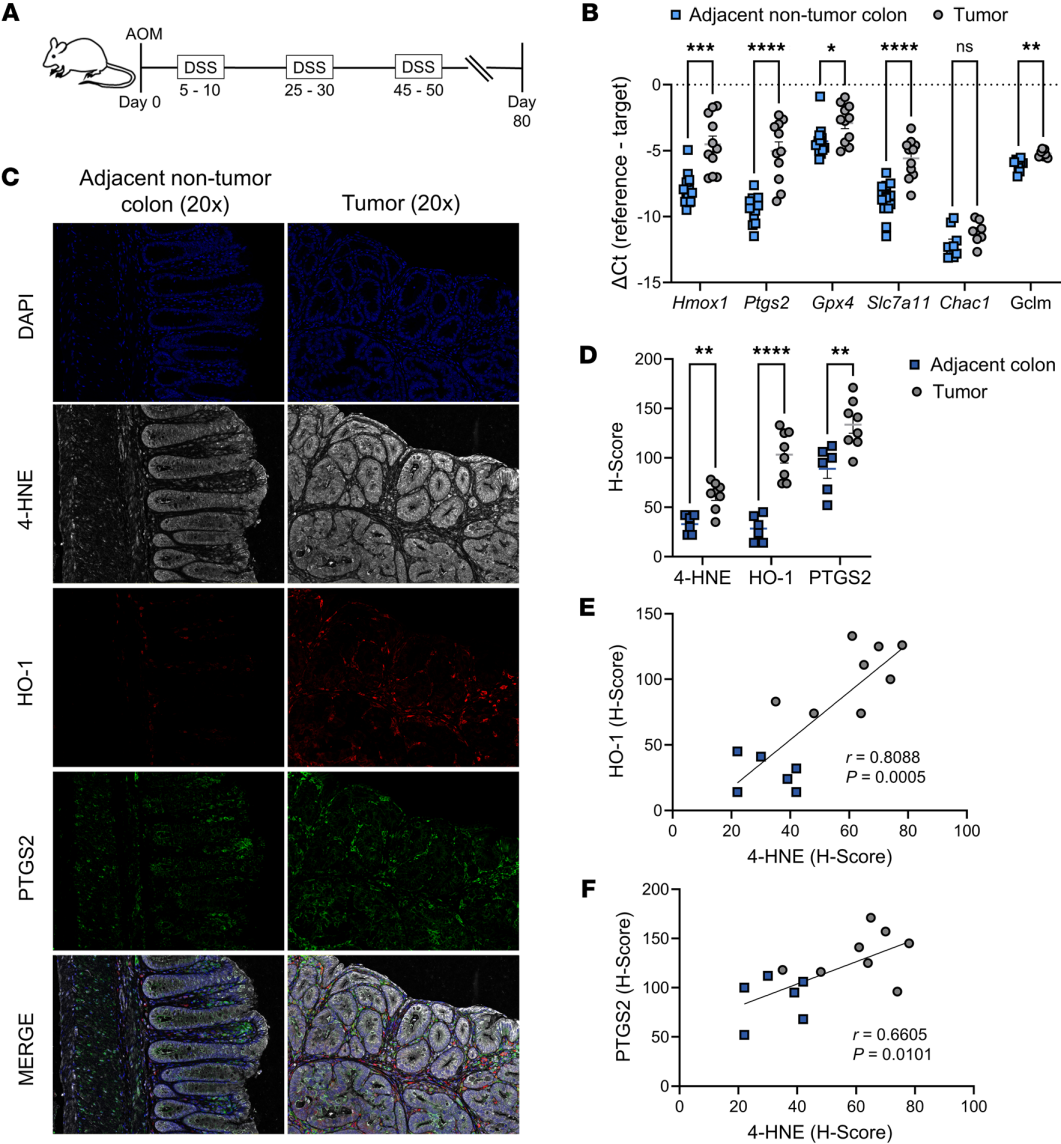
HO-1, lipid peroxidation, and ferroptosis response genes are upregulated in colonic tumors. (**A**) Schematic representation of the azoxymethane-DSS colitis-associated cancer model. (**B**) RT-qPCR analysis of tumors (pooled per animal, n = 7 – 11) and uninvolved colon (n = 9 – 14) from WT C57BL/6 mice in the AOM-DSS model. (**C**) Representative photomicrographs of immunofluorescence staining using paraffin-embedded mouse tumor and adjacent colon sections from an AOM-DSS colitis experiment (original magnification, ×20). (**D**) Histologic scores of proteins from individual tumor sections from 8 mice and adjacent colon sections from 6 mice. (**E** and **F**) Pearson correlations comparing the lipid peroxidation byproduct 4-HNE to either HO-1 or PTGS2. Data are shown as the mean ± SEM. *P < 0.05, **P < 0.01, ***P < 0.001, and ****P < 0.0001, by multiple unpaired Student’s *t* tests with correction for multiple comparisons (colon vs. tumor for each gene).

To investigate the role of HO-1 in this context we employed the azoxymethane-DSS (AOM-DSS) model of CAC (Figure 3A), which models chronic epithelial injury leading to the development of colonic tumors (37). Using WT mice, we observed significantly increased expression of ferroptosis-response genes in the colon tumors, compared with adjacent uninvolved colon, including *Hmox1*, *Ptgs2*, and *Slc7a11* (Figure 3B). These findings suggest that the tumor microenvironment (TME) is characterized by elevated oxidative stress and lipid peroxidation.

To validate these transcriptional changes at the protein level, we performed quantitative multiplexed immunofluorescence using the PhenoImager HT platform. We observed significantly increased levels of PTGS2, 4-HNE, and HO-1 in tumor sections compared with matched nontumor colon tissue (Figure 3, C and D). Notably, 4-HNE levels strongly correlated with detection of HO-1 and PTGS2, reinforcing the link between HO-1 induction and oxidative lipid damage within the TME (Figure 3, E and F). Collectively, these data support increased lipid peroxidation in the TME while the consistent and strong association with HO-1 suggests it could be a biomarker of oxidative stress activity in colitis-associated tumors and warrants further investigation of a potential mechanistic role.

### Deletion of epithelial HO-1 reduces tumor burden and colitis-associated tumorigenesis.

**Figure 4:**
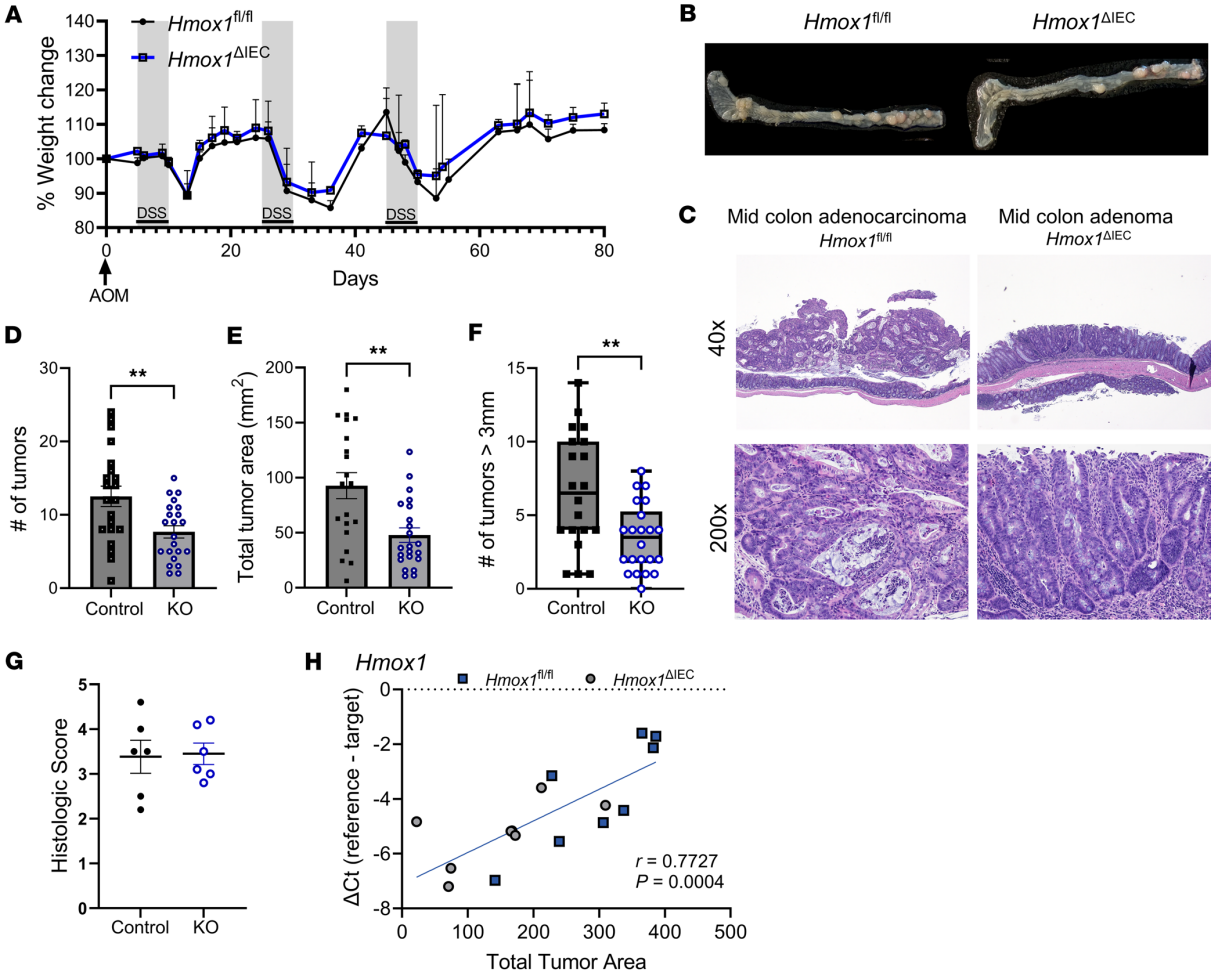
Epithelial HO-1 deletion reduces tumor burden in colitis-associated cancer. (**A**) Comparison of body weight changes over the course of the AOM-DSS colitis experiment in *Hmox1*^fl/fl^ and *Hmox1*^ΔIEC^ mice (*n* = 8–11 mice per group, pooled from 2 independent experiments). (**B**) Representative colons with demonstration of tumor formation at day 80 from both mouse strains. (**C**) Representative H&E staining of sections of colonic tumors from the AOM-DSS model (original magnification, ×40 [top]; ×200 [bottom]). (**D**) Visible tumor numbers in colon and cecum at day 80 upon gross examination. Combined data from 3 independent experiments. (**E**) Tumor burden and average size, as measured by 2D area of tumor involvement. (**F**) Number of tumors >3 mm in diameter visualized in colon and cecum at day 80 upon gross examination. (**G**) Colonic histopathologic injury score at day 80. (**H**) Pearson correlation of pooled whole tumor *Hmox1* mRNA expression (qPCR) with tumor burden. Data are shown as the mean ± SEM. *P <.05, **P <.01, by unpaired, Student’s *t* test.

Given the consistent upregulation of HO-1 in colonic tumors and its association with oxidative stress, we next investigated whether epithelial HO-1 plays a functional role in colitis-associated tumorigenesis. Using the AOM-DSS model, we compared tumor development in our control *Hmox1*^fl/fl^ mice and our *Hmox1*^ΔIEC^ mice during the course of chronic colitis. We observed similar weight loss, bleeding scores and fecal heme content between the 2 groups (Figure 4A and Supplemental Figure 3). However, *Hmox1*^ΔIEC^ mice developed significantly fewer colonic tumors compared with controls (Figure 4, B–D). Tumors in KO mice were predominantly located in the distal colon, consistent with known injury patterns in this model (Figure 4B and Supplemental Figure 4A) (23). Both adenomas and adenocarcinomas were observed (Figure 4C and Supplemental Figure 4B). Tumor burden, measured by total surface area and estimated volume, was significantly reduced in *Hmox1*^ΔIEC^ mice, and large tumors (>3 mm) were less frequent (Figure 4, E and F, and Supplemental Figure 4, C and D). The number of colonic tumors was similarly decreased in both male and female *Hmox1*^ΔIEC^ mice (Supplemental Figure 4E). Importantly, histologic injury scores during DSS colitis and at the endpoint were comparable between genotypes, indicating that reduced tumor burden was not due to differences in inflammation severity (Figure 4G and Supplemental Figure 3E).

To further explore the relationship between HO-1 and tumorigenesis, we analyzed *Hmox1* mRNA expression in colonic tissue and assessed its correlation with tumor burden across both control and KO mice. A strong positive correlation was observed between *Hmox1* expression and total tumor area and volume, supporting the hypothesis that elevated HO-1 may be associated with enhanced tumor growth (Figure 4H). Notably, *Hmox1* expression levels in KO mice were not uniformly the lowest, likely reflecting contributions from nonepithelial cell types such as immune or stromal cells, suggesting further complexity in cell type–specific roles for HO-1 in tumorigenesis.

Collectively, these results provide functional evidence that epithelial HO-1 influences tumor development in CAC. Given prior findings linking HO-1 to regulation of oxidative stress responses, we next examined the consequences of HO-1 deletion on oxidative damage within the TME.

### Epithelial HO-1 regulates iron availability and tumor epithelial transcriptional adaptation to oxidative stress.

**Figure 5:**
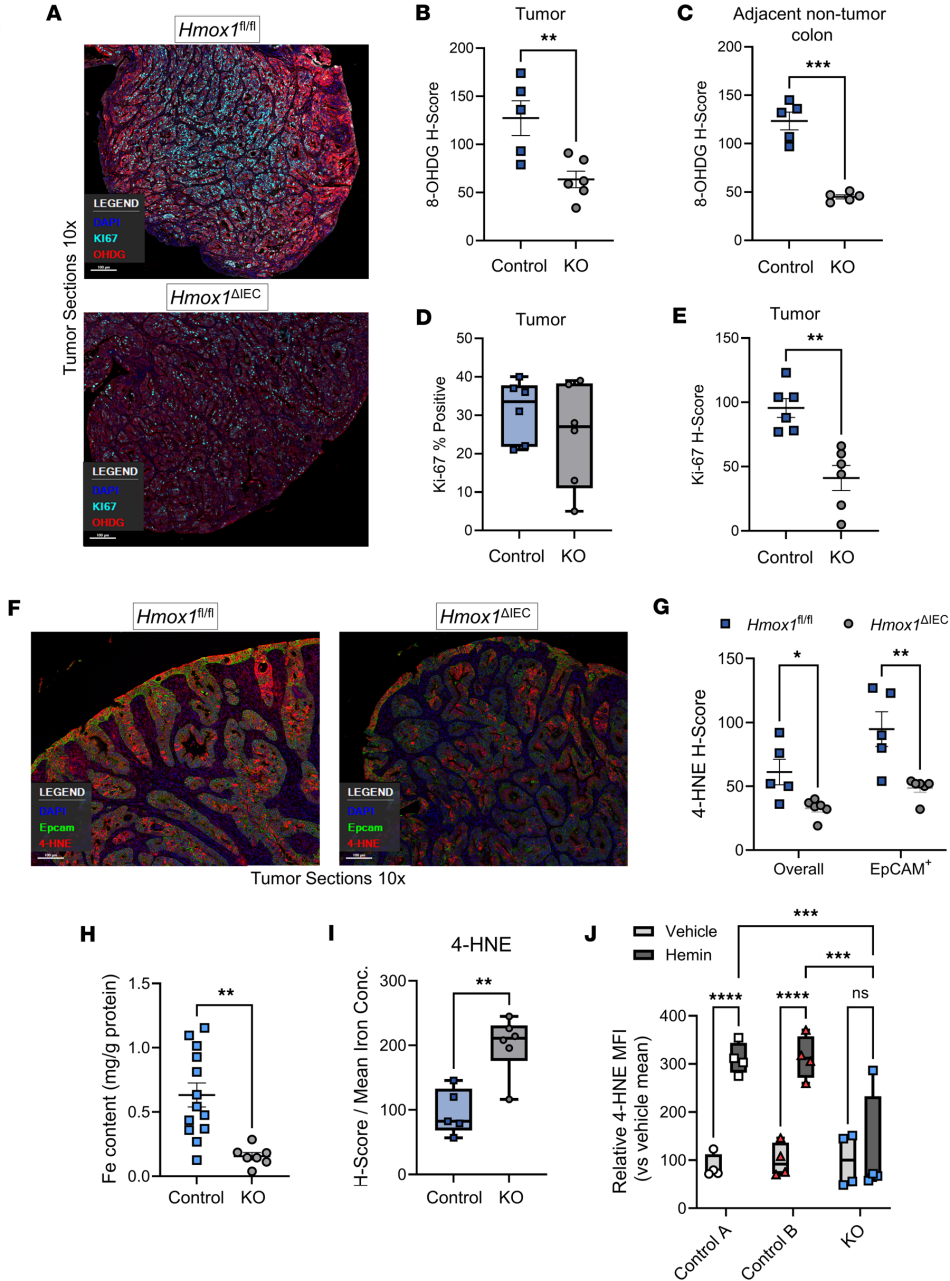
Epithelial HO-1 regulates oxidative damage, iron levels, and stress adaptation in the TME. (**A**) Representative photomicrographs of immunofluorescence staining for 8-hydroxy-2′-deoxyguanosine (8-OHdG) and Ki-67, using paraffin-embedded mouse colonic individual tumor sections from 4 control and 4 *Hmox1*^ΔIEC^ mice undergoing AOM-DSS (original magnification, ×10). (**B**) Quantification of 8-OHdG staining intensity in tumor tissue. (**C**) 8-OHdG staining intensity in adjacent nontumor colonic tissue from control and KO mice. (**D**) Percentage of Ki-67^+^ cells based on immunofluorescence staining. (**E**) Ki-67 H-score analysis from tumor sections. (**F**) Representative photomicrographs of immunofluorescence staining for 4-HNE and EpCAM using colon tumor sections (original magnification, ×10). (**G**) Quantification of 4-HNE from tumor sections taken from control and KO mouse tumors. (**H**) Whole tumor iron concentration measured by colorimetric assay. (**I**) 4-HNE staining intensity normalized to mean tumor iron levels per genotype. (**J**) Tumoroids derived from pooled tumors from 2 *Hmox1*^fl/fl^ mice (control **A** and **B**) and 1 *Hmox1*^ΔIEC^ (KO) mice were exposed to hemin (100 μM) for 24 hours. Lipid peroxidation measured by flow cytometry for 4-HNE, with median fluorescence intensity (MFI) values normalized to the mean of each vehicle-treated group. Data are shown as the mean ± SEM. *P < 0.05, **P < 0.01, ***P < 0.001, and ****P < 0.0001 by unpaired, Student’s *t* tests (**B**-**E,** **G**-**I**) and ANOVA (**J**) with correction for multiple comparisons.

To investigate the impact of epithelial HO-1 deletion on oxidative stress and its influence on tumor epithelial proliferation in our CAC model, we stained for 8-hydroxy-2′-deoxyguanosine (8-OHdG), which measures oxidative DNA damage, and Ki-67, a marker of proliferation, alongside DAPI and EPCAM. Tumors from KO mice exhibited significantly reduced 8-OHdG staining compared with controls (Figure 5, A and B). Interestingly, this reduction was also observed in adjacent nontumor colonic tissue (Figure 5C), suggesting that epithelial HO-1 contributes to oxidative DNA damage in chronically inflamed and neoplastic tissue. Ki-67 staining revealed a modest, nonsignificant reduction in the percentage of proliferating cells in KO tumors (Figure 5D). However, Ki-67 expression levels, as measured by H-score, were significantly lower in *Hmox1*^ΔIEC^ tumors (Figure 5E), indicating reduced proliferative activity. These findings support a role for epithelial HO-1 in promoting tumor cell proliferation in CAC, possibly by regulating oxidative stress and damage to DNA.

To explore the relationship between HO-1 deletion and lipid peroxidation, we quantified 4-HNE staining in tumor sections. Representative images and H-score analysis of whole tumors and EPCAM^+^ epithelial cells showed significantly reduced 4-HNE staining in tumors from *Hmox1*^ΔIEC^ mice compared with controls (Figure 5, F and G). A previous study demonstrated that overexpression of HO-1 in a colorectal cancer cell line increases the labile iron pool and promotes lipid peroxidation (38). We therefore measured tumor iron content to determine whether loss of epithelial HO-1 influences lipid peroxidation via regulation of iron levels. We measured significantly decreased iron in tumors from *Hmox1*^ΔIEC^ mice (Figure 5H), consistent with expectations that impaired heme catabolism can reduce labile iron levels and influence iron-dependent oxidative damage. Paradoxically, when tumor 4-HNE staining was normalized to average (mean) iron content by strain, KO tumors showed significantly higher 4-HNE per unit iron (Figure 5I), suggesting increased iron-independent oxidative lipid damage. This could reflect oxidative damage driven by uncatabolized heme, rather than free iron, consistent with known oxidative and nonoxidative mechanisms of heme toxicity (22, 39).

As colonic tumors grow, increased and often dysregulated vascularity can lead to microhemorrhages and cell death, resulting in elevated levels of heme, hemoglobin, and hemeproteins (40). To further validate the influence of epithelial HO-1 in regulating tumor epithelial responses to heme within the TME, as a proof of concept, we developed tumor epithelium cell-derived organoids (tumoroids) from 2 control (*Hmox1*^fl/fl^) mice and 1 KO (*Hmox1*^ΔIEC^) mouse. After exposure to hemin, control tumoroids demonstrated significantly elevated 4-HNE levels compared with vehicle-treated controls, consistent with HO-1–mediated iron release causing lipid peroxidation (Figure 5J). In contrast, KO tumoroids showed no increase in 4-HNE after exposure to hemin (Figure 5J), supporting a requirement for HO-1 in hemin-induced lipid peroxidation in tumor epithelial cells. These results reinforce the role of HO-1 in shaping epithelial oxidative stress responses in the TME.

### Single-cell transcriptomic profiling reveals stress-adaptive epithelial responses.

**Figure 6:**
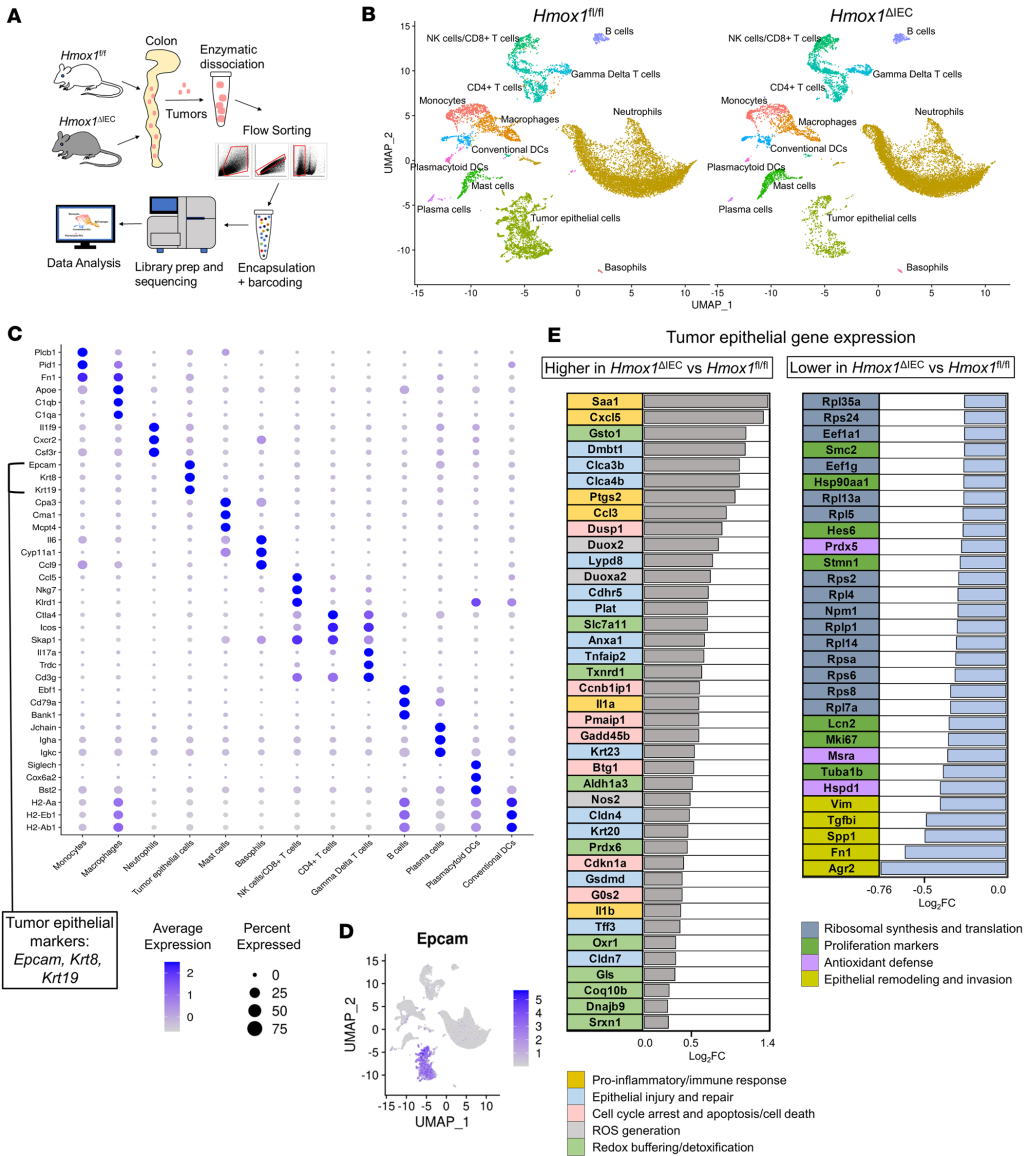
Loss of epithelial HO-1 induces a stress-adaptive transcriptional shift in tumor epithelial cells. (**A**) Overview for single-cell RNA sequencing workflow. Pooled tumors from control and KO mice were enzymatically dissociated and live cells were sorted for scRNA-seq using the 10X Genomics platform. (**B**) UMAP plot of approximately 24,000 quality-controlled cells from both genotypes, showing unsupervised clustering and manual annotation of major cell types. (**C**) Dot plot of top 3 marker genes per cluster used to validate annotations, ranked according to log_2_ fold change multiplied by difference in percentage expression compared with all other cells. (**D**) Feature plot of *Epcam* expression across the UMAP, highlighting the epithelial subcluster used for differential expression analysis. (**E**) Bar graphs of significantly upregulated and downregulated genes in *Epcam*^+^ epithelial cells from KO tumors, identified using the MAST hurdle model with Bonferroni-adjusted *P* < 0.05 and log_2_FC > 0.25. Genes are color-coded by functional annotation.

Given the potential for noncatabolized heme to induce cellular stress independent of iron, we further explored how loss of HO-1 influences epithelial cell stress responses within the TME by performing single-cell RNA sequencing (scRNA-seq). Pooled tumors from *Hmox1*^fl/fl^ and *Hmox1*^ΔIEC^ mice >2 mm in diameter were enzymatically dissociated, and live cells were sorted for sequencing using the 10X Genomics platform (Figure 6A). After quality control, library normalization, and dimensionality reduction, approximately 24,000 cells were coembedded in UMAP space (Figure 6B). Unsupervised clustering initially identified 18 distinct populations, which were annotated using a combination of canonical marker genes (e.g., T cells, *CD3e*; B cells, *CD79a*; tumor epithelial cells, *Epcam*; neutrophils, *Cxcr2*; monocyte/macrophages: *Fcgr1*; mast cells, *Fcer1a*) and guidance from prior scRNA-seq datasets from AOM-DSS and murine intestine (Figure 6C and Supplemental Figure 5) (41–43). Both genotypes exhibited similar cellular composition, including tumor epithelial cells and diverse leukocyte populations (Figure 6B).

Analysis of the *Epcam*^+^ tumor epithelial cell cluster (Figure 6D) revealed distinct transcriptional programs between genotypes (Figure 6E). In KO epithelial cells, a reactive and stress-adaptive phenotype was observed, marked by oxidative stress responses, heightened inflammation, cell cycle regulation, and stress-induced programmed cell death. This profile is driven by a robust oxidative stress response, with increased expression of genes involved in ROS generation (*Duox2*, *Duoxa2*, *Nos2*) and redox buffering/detoxification (*Txnrd1*, *Gsto1*, *Aldh1a3*, *Slc7a11*, *Prdx6*, *Oxr1*, *Dnajb9*), consistent with efforts to mitigate oxidative damage and maintain cellular homeostasis. This transcriptional shift is consistent with an adaptive response to toxicity from uncatabolized heme and aligns with the stress-adaptive phenotype observed in vitro (22). In line with this, genes regulating cell cycle arrest and apoptosis (*Cdkn1a*, *Ccnb1ip1*, *Gadd45b*, *Btg1*, *Pmaip1*, *Gsdmd*, *Dusp1*) were elevated, reflecting a tightly controlled response to cellular stress. Elevated expression of genes involved in proinflammatory and immune responses (*Saa1*, *Cxcl5*, *Ccl3*, *Il1a*, *Il1b*, *Ptgs2*) was also observed. Genes associated with epithelial injury and repair (*Dmbt1*, *Lypd8*, *Clca3b*, *Clca4b*, *Plat*, *Anxa1*, *Tnfaip2*, *Krt20*, *Krt23*, *Cldn4*, *Cdhr5*) were also upregulated, suggesting active tissue repair/remodeling.

In contrast, control epithelial cells exhibited a transcriptional profile consistent with a more aggressive, proliferative, and invasive TME. These cells upregulated genes linked to iron metabolism and metastatic potential (*Lcn2*), epithelial remodeling and invasion (*Tgfbi, Spp1, Agr2, Fn1, Vim*), and general antioxidant defense (*Prdx5, Msra, Hspd1*). Elevated expression of proliferation markers such as *Mki67, Hsp90aa1, Tuba1b* and *Hist1h4c* aligns with increased Ki-67 staining, supporting a more proliferative tumor phenotype.

## Discussion

Colitis-associated colorectal cancer arises in the context of chronic mucosal inflammation, epithelial injury, and hemorrhage, which together create a prooxidant microenvironment rich in luminal heme. While epithelial HO-1 is traditionally viewed as cytoprotective, our findings reveal a context-dependent role in tumorigenesis. Specifically, epithelial HO-1 protects against *acute* heme toxicity (8, 12) but paradoxically promotes tumor growth in *chronic* inflammation by increasing intracellular iron availability and amplifying oxidative damage.

Our data demonstrate that HO-1 is consistently upregulated in colonic epithelium during active colitis and in tumors, correlating with measures of oxidative stress (31–34). Beyond mechanisms driven by iron released through HO-1–mediated catabolism, intact heme itself can exert cytotoxic effects through multiple pathways. These include intercalating into lipid membranes, proteasome inhibition, disrupting membrane integrity, generating ROS without iron release, and damaging intracellular organelles via both oxidative and nonoxidative mechanisms (22, 39, 44). These alternative modes of heme-induced injury may be particularly relevant in the absence of HO-1, which normally facilitates heme detoxification through catabolism and iron sequestration. The cytoprotective role of HO-1 under acute heme stress was evident in our colonoid experiments where heme exposure to HO-1–deficient colonoids led to increased cell death, cell cycle arrest, and a robust stress-adaptive transcriptional response designed to compensate for lost antioxidative and detoxification function (8, 28, 29). In chronically inflamed colonic epithelium of *Hmox1*^ΔIEC^ mice this compensatory response, along with regulation of iron availability, may influence tumor initiation by reducing oxidative DNA damage. In line with this, in *Hmox1*^ΔIEC^ mice, we observed a significant reduction in tumor number and size, despite comparable levels of colitis-induced injury. This suggests that epithelial HO-1 contributes directly to tumorigenesis, independent of inflammation severity, in contrast to many studies on CAC (37). Mechanistically, HO-1 deletion led to reduced oxidative DNA damage (8-OHdG), diminished 4-HNE accumulation as a product of lipid oxidation, and lower iron levels in tumors (34, 38). These findings also support a model in which epithelial HO-1–mediated heme catabolism augments tumor growth by increasing bioavailable iron and driving proliferation, despite fueling oxidative stress and damage (13, 20, 21). Iron promotes tumor growth by supporting nucleotide metabolism and proliferation (20, 21), positioning HO-1 as a key upstream regulator of iron-dependent metabolic reprogramming in the TME. In its absence, reduced iron availability and the compensatory stress response may jointly suppress tumor progression. Interestingly, when 4-HNE was normalized to iron content, KO tumors exhibited higher 4-HNE per unit iron. This suggests a less potent source of oxidative damage, independent of free iron, likely due to uncatabolized heme (22).

scRNA-seq revealed that tumor epithelial cells from *Hmox1*^ΔIEC^ mice adopt a stress-adaptive transcriptional program characterized by upregulation of redox buffering, cell cycle arrest, and programmed cell death pathways. This stress-adaptive phenotype, likely driven by persistent heme toxicity, may suppress tumor progression by limiting proliferation and promoting repair. In contrast, control tumor epithelial cells exhibited a more proliferative and invasive phenotype, with elevated expression of iron metabolism genes and markers of epithelial-mesenchymal transition. These transcriptional differences mirror the histologic and molecular phenotypes, reinforcing HO-1’s role in promoting a proliferative tumor cell state.

Our findings contribute to the growing literature on iron-dependent oxidative stress and lipid peroxidation in gastrointestinal cancers. Ferroptosis, driven by iron-dependent lipid peroxidation, represents one potential outcome of dysregulated iron metabolism, and HO-1 has been reported to both promote and suppress ferroptosis, depending on cellular context and iron availability (45–50). However, our data primarily support a contextual role for HO-1 in regulating oxidative damage and proliferation through regulation of iron availability. In colonic epithelial cells exposed to heme, HO-1 appears protective, limiting cell death and oxidative damage (8, 29). However, in the TME, HO-1 may facilitate iron accumulation and oxidative stress, promoting proliferation and tumor progression (20, 38). This duality has important implications. Therapeutic strategies targeting HO-1 or iron metabolism must consider the temporal and spatial context of HO-1 activity. Inhibiting HO-1 may sensitize tumor cells to ferroptosis or constrain proliferation by limiting iron availability but could also impair epithelial resilience to acute heme stress (51). Conversely, enhancing HO-1 activity may protect against inflammation-induced damage but risk promoting tumorigenesis in chronic disease.

In summary, our study identifies epithelial HO-1 as a key regulator of iron metabolism, oxidative stress, and tumor epithelial cell adaptation in CAC. The apparent context-dependent function of HO-1, cytoprotection in acute injury but tumor promotion in chronic inflammation, highlights its complex role in colonic epithelial homeostasis and neoplasia. Future studies could further explore the therapeutic implications of manipulating epithelial HO-1 and lipid peroxidation in other models of inflammatory bowel disease and colorectal cancer.

## Methods

### Sex as a biological variable

Our study examined male and female mice, and similar findings are reported for both sexes.

### DSS colitis versus AOM-DSS colitis

C57BL/6J WT mice were used where indicated and C57BL/6 *Hmox1*^fl/fl^ mice were provided by H.B. Suliman, Duke University, Durham, North Carolina (52). *Hmox1*^ΔIEC^ mice were made by crossing *Hmox1*^fl/fl^ mice with B6.Cg-Tg(Vil1-cre)1000Gum/J mice expressing Cre recombinase in villus and crypt epithelial cells of the small and large intestines (JAX, 021504) (53). Littermate controls were used for all colitis experiments. We used age-matched male and female mice starting between age 7 and 14 weeks. For colitis experiments, mice were given 2.5% colitis-grade DSS (MP Biomedical) in drinking water, changed every 2 days, for 5 days and were then sacrificed at day 7 where indicated. For the CAC model, a single dose of AOM at 10 mg/kg of body weight was administered intraperitoneally on day 0, and 5 days later, mice underwent 3 rounds of 5 days of 2.5% DSS in drinking water, followed by 14 days of tap water, with the final period of tap water extended to 30 days (Figure 3A). Mice were sacrificed on day 80. Disease activity components were measured blindly (weight change, stool consistency, and hematochezia scores; scale 0–3) and an index was computed by summing the scores (maximum score of 9). At experiment end, colons were excised, cleaned, the lumen was then exposed, and photos were taken with ruler or calipers for measurements of the tumors. Final tumor measurements (e.g., area) were calculated using ImageJ (NIH) (54). Colons were fixed, embedded, and processed for histology. H&E-stained tissue was scored by a histopathologist blinded to the treatments and groupings of animals using described methods (23).

### IEC/leukocyte isolation and flow cytometry

Isolation of tumor cells and intestinal epithelial cells was performed as previously described (55). Modifications include retention of the dissociated epithelial fraction where necessary for FACS and measurement of *Hmox1* expression. We performed flow cytometry as previously described (55). Dead cells were stained and excluded using BioLegend 7AAD viability staining solution or Zombie Aqua dye prior to flow cytometry or FACS. Anti-4-HNE (R&D Systems, MAB3249) was used with an AF647 secondary antibody (Invitrogen) to detect lipid peroxidation via flow cytometry, which was validated using H_2_O_2_ as a positive control. Other anti-mouse antibodies were purchased from BioLegend or Thermo Fisher Scientific: CD45 (30-F11) and EpCAM (G8.8). Flow cytometry was performed on a FACSCanto II (Becton Dickinson) or a BD FACSAria Fusion, and resulting data were analyzed with De Novo’s FCS Express 7 software.

### Cell culture

Epithelial cells were harvested from 8- to 12-week-old *Hmox1*^fl/fl^ and *Hmox1*^ΔIEC^ mouse colons or tumors and processed as previously described to make colonoids (56). Tumor-derived organoids were made from pooled tumors from 2 control mice separately and 1 KO mouse. Data are representative of 2–3 independent experiments with identical results. Human colonic epithelial organoids were obtained by biopsy via colonoscopy. Organoids were maintained in L-WRN cell-conditioned enteroid media (DMEM/F12 supplemented with 1% Pen/Strep, 1% glutamax, 10% FBS, and with ROCK inhibitor Y-27632) in a humidified atmosphere with 5% CO_2_ at 37°C. Organoids were cultured for 7–10 days, passaged, and used typically 48 hours after plating. Organoids were also frozen and used after successful thawing. Hemin was purchased from Sigma-Aldrich. Erastin was purchased from MedChemExpress.

### Reverse transcription quantitative PCR and mRNA sequencing

RNA was isolated from snap-frozen tissue samples or cells using Trizol (Invitrogen) or Qiagen RNeasy Mini kit, respectively, with the addition of the gDNA-eliminator columns as per the manufacturers’ protocol. First-strand complementary DNA synthesis was performed using iScript reverse transcription super-mix from Bio-Rad. Real-time quantitative PCR (qPCR) was performed in technical duplicates using SYBR green on Applied Biosystems QuantStudio 5 Real-Time PCR System. Due to variability in transcript detection across samples, the number of biological replicates included in an analysis may differ by gene. β-Actin (*Actb*) was used as the reference gene and results are reported as ΔCt, which is the Ct of the target gene subtracted from the Ct of the reference gene (higher ΔCt values = higher expression relative to the reference gene). Primer sets were purchased from Sigma-Aldrich’s KiCqStart line. Preparation of mRNA library, sequencing, and bioinformatic analysis was conducted by Novogene Corporation Inc.

### Heme quantification assay

Total heme was measured using an established protocol with minor adaptations (57).

### Intracellular iron quantification

Organoids were suspended and lysed in RIPA buffer. The Pierce BCA Protein Assay Kit was used to obtain total protein concentration of organoids. The Iron Assay Kit (Colorimetric) from Novus Biologicals was used to measure total iron content. Organoid lysate was substituted for tissue homogenate in the manufacturer’s protocol. Both the BCA and Iron assay were measured using a BioTek Synergy H1 microplate reader. Results were analyzed using Microsoft Excel.

### Quantitative multiplexed immunofluorescence

Through our collaboration with the Human Immune Monitoring Shared Resource at the University of Colorado School of Medicine, we performed multispectral imaging using the PhenoImager HT instrument (formerly Vectra Polaris, Akoya Biosciences). Deparaffinized FFPE tissue sections were heat treated in antigen retrieval buffer, blocked, and incubated with various primary antibodies, including HO-1 (Abcam, ab189491; 1:1,000; pH9), PTGS2 (Cell Marque; 1:50; pH6), 4-HNE (Abcam, ab46545; 1:100; pH6), 8-OHdG (BioSS, bs-1278R; 1:300; pH9), Ki67 (SP6, 1:400; pH6), and EPCAM (1:200; pH6), followed by horseradish peroxidase–conjugated secondary antibody polymer and HRP-reactive OPAL fluorescent reagents. Slides were stripped between each stain with heat treatment in antigen retrieval buffer. Whole slides were imaged with PhenoImager HT v2.0.0, ×20 objective, 0.5-micron resolution. Images were then analyzed using inForm software v3.0 (Akoya Biosciences).

### Western blot

Total protein content was obtained using the Pierce BCA Protein Assay Kit. 10% Mini-PROTEAN TGX Precast Protein Gel from Bio-Rad was used to separate proteins in 10% SDS-PAGE. Using the Trans-Blot Turbo RTA Midi 0.2 μm PVDF Transfer Kit from Bio-Rad, proteins were transferred onto membranes in the kit. Membranes were blocked with 5% nonfat milk blocking buffer and then incubated overnight at 4°C with primary antibody 4-HNE (1:1,000) from Abcam. The control used was β-tubulin (Sigma-Aldrich, T4026). Then, blots were stained with secondary antibody. Protein bands were enhanced using Clarity Western ECL Substrate from Bio-Rad and analyzed using Bio-Rad ChemiDoc MP Imaging System.

### Human colonic biopsy tissue

Patient colon biopsy samples were collected during colonoscopy and made available as part of a biobank repository at the University of Colorado Anschutz Medical Campus Crohn’s and Colitis Center with Colorado Multiple Institutional Review Board approval. ELISA for HO-1 was performed using a human HO-1 DuoSet IC kit from R&D Systems, and total protein was assessed using a Pierce BCA Protein Assay Kit.

### NCBI Gene Expression Omnibus

In data set GSE38713, colonic gene expression in patients with UC and individuals acting as noninflammatory controls was assayed using Affymetrix GeneChip high-density oligonucleotide HGU133 Plus 2.0 microarrays (30). Initial analysis was performed using GEO2R with log_2_ transformation, and *P* values were adjusted using the Benjamini and Hochberg FDR method (58). Only probe sets that represent a single gene (labeled as *_at) were used.

### scRNA-seq

Pooled tumors from each of 2 *Hmox1*^fl/fl^ and 2 *Hmox1*^ΔIEC^ mice were used. Cells were enzymatically dissociated, and total live cells were recovered by FACS (55). Cell capture, sequencing, and library construction were performed with the assistance of the University of Colorado Genomics Shared Resource (Next GEM 3′ Gene Expression v3, 10X Genomics).

#### Processing.

Processing and statistical analyses were performed in R version 4.2. (59) and Seurat version 4.3.0 (60). Seurat defaults were used unless otherwise noted. The data were restricted to features with at least 3 detected cells, and low-quality cells with more than 15% mitochondrial genes or less than 300 genes were filtered out. The DoubletFinder package version 2.0.3 (61) was then used to remove multiplets. This yielded 23,698 high-quality cells across all of the samples.

Library size normalization was performed with NormalizeData, and the top 2000 highly variable genes were identified with the FindVariableFeatures variance stabilizing transformation method. Features that were repeatedly variable across datasets were then used as anchors to create an integrated dataset across samples with the FindIntegrationAnchors and IntegrateData functions. Dimensionality reduction was performed for visualization; the integrated data were scaled and centered with ScaleData, and principal component analysis was performed with RunPCA to reduce the data to 30 principal components (PCs). RunUMAP was then run on the PCs.

#### Unsupervised clustering and annotation.

The FindClusters algorithm was run on the PCs with a resolution of 0.45 to identify clusters in the overall cell population. Subclustering of epithelial (resolution = 0.038) cell populations was analogously performed. The DimPlot function was then used to create UMAPs stratified by treatment group. Positive conserved cell-type markers were identified using the FindAllMarkers function with minimum detection and log_2_-fold change thresholds set to 25% and Bonferroni-adjusted *P* ≤ 0.05. The markers were used to perform manual annotation and to merge and split clusters when appropriate. The marker genes were sorted by the log_2_-fold change multiplied by the difference in percentage of cells expressed between the cluster and all other cells. The top marker genes were then visualized with Dotplots (ggplot2 package version 3.4.0, ref. 62), feature plots (FeaturePlot), and heatmaps (ComplexHeatmap package version 2.12.1, ref. 63).

#### Gene ontology enrichment analysis of epithelial cell populations.

Epithelial subpopulation differentially expressed genes (DEGs) between *Hmox1*^fl/fl^ and *Hmox1*^ΔIEC^ mice were identified using the FindAllMarkers MAST Hurdle Approach (64) and previously described thresholds (see above). Genes were considered differentially expressed if Bonferroni-adjusted significance was less than 0.05. DEGs were then analyzed to determine whether they were enriched relative to all genes in the dataset. Genes were first annotated to Gene ontology terms with the biomaRt R package (65), and genes without annotations were eliminated.

### Statistics

Unless otherwise stated, statistical significance of comparisons was performed using Prism version 9.5 and 10 (GraphPad Software). We used unpaired two-tailed Student’s *t* tests for two groups with correction for multiple comparisons using the Holm-Šidák method, or ANOVA for multiple groups followed by Bonferroni or Dunnett’s post hoc test. Error bars reflect SEM unless otherwise stated. Results were considered statistically significant if *P* < 0.05.

### Study approval

All animal studies were performed in accordance with protocols approved by the University of Colorado Anschutz Medical Campus and Rocky Mountain Regional VAMC Institutional Animal Care and Use Committees. Patient colon biopsy samples were collected during colonoscopy and made available as part of a biobank repository at the University of Colorado Anschutz Medical Campus Crohn’s and Colitis Center with Colorado Multiple Institutional Review Board approval.

### Data availability

All data and methods supporting the findings of this study are available within the paper and its supplemental materials. The bulk RNA-seq data are available under GEO GSE312912. The single-cell RNA-seq data are available under GEO GSE312733. Underlying data for the figures are available in the Supporting Data Values file. Data are also available upon request.

## Author contributions

The study was designed and supervised by JCO with the support of SPC. RCC and JCC performed most of the experiments/data acquisition, with contributions from GB, REMS, FM, LZ, JCO, RMN, CHTH, CAS, MEG, and IMC. JCO and AWS (bioinformatics) performed data analysis and visualization with contributions from DJO, RCC, GB, and SMA. Resources and funding were provided by JCO and SPC, with contributions from CHTH, ALT, CAS, and MEG. JCO, RCC, JCC, RMN, CHTH, GB, FM, LZ, and REMS contributed to methodology. Important intellectual contributions were provided by SPC, SMA, and IMC. The original manuscript was written by JCO with contributions from RCC, JCC, and AWS. Review and editing of the manuscript was by SPC, SMA, JCO, CHTH, AWS, DJO, RCC, ALT, and IMC, with input from all coauthors. RCC and JCC contributed equally and share co–first authorship. The order of appearance was determined by their chronological involvement with the project.

## Funding support

This work is the result of NIH funding, in whole or in part, and is subject to the NIH Public Access Policy. Through acceptance of this federal funding, the NIH has been given a right to make the work publicly available in PubMed Central.

- US Department of Veterans Affairs BLR&D Service Career Development Award (BX003865 to JCO).
- University of Colorado GI and Liver Innate Immune Program pilot award (to JCO).
- Merit Reviews from the US Department of Veterans Affairs BLR&D Service (BX002182 to SPC and BX005288 to ALT).
- NIH R01 DK50189 (to SPC).
- Genomics Shared Resource/Cancer Center Support Grant (P30CA046934).

## Supplementary Material

Supplemental data

Unedited blot and gel images

Supporting data values

## Version 1 12/18/2025

In-Press Preview

## Version 2 02/09/2026

Electronic publication

## References

[1] Olen O (2020). Colorectal cancer in ulcerative colitis: a Scandinavian population-based cohort study. Lancet.

[2] Jess T (2012). Risk of colorectal cancer in patients with ulcerative colitis: a meta-analysis of population-based cohort studies. Clin Gastroenterol Hepatol.

[3] Aardoom MA (2018). Malignancy and mortality in pediatric-onset inflammatory bowel disease: a systematic review. Inflamm Bowel Dis.

[4] Ekbom A (1990). Ulcerative colitis and colorectal cancer. A population-based study. N Engl J Med.

[5] Schroeder KW (1987). Coated oral 5-aminosalicylic acid therapy for mildly to moderately active ulcerative colitis. A randomized study. N Engl J Med.

[6] D’Haens G (2007). A review of activity indices and efficacy end points for clinical trials of medical therapy in adults with ulcerative colitis. Gastroenterology.

[7] Glei M (2006). Hemoglobin and hemin induce DNA damage in human colon tumor cells HT29 clone 19A and in primary human colonocytes. Mutat Res.

[8] Seiwert N (2020). Heme oxygenase 1 protects human colonocytes against ROS formation, oxidative DNA damage and cytotoxicity induced by heme iron, but not inorganic iron. Cell Death Dis.

[9] Canesin G (2020). Scavenging of labile heme by hemopexin is a key checkpoint in cancer growth and metastases. Cell Rep.

[10] Seiwert N (2021). Chronic intestinal inflammation drives colorectal tumor formation triggered by dietary heme iron in vivo. Arch Toxicol.

[11] Smith A, McCulloh RJ (2015). Hemopexin and haptoglobin: allies against heme toxicity from hemoglobin not contenders. Front Physiol.

[12] Ryter SW (2006). Heme oxygenase-1/carbon monoxide: from basic science to therapeutic applications. Physiol Rev.

[13] Poss KD, Tonegawa S (1997). Heme oxygenase 1 is required for mammalian iron reutilization. Proc Natl Acad Sci U S A.

[14] Radhakrishnan N (2011). Human heme oxygenase-1 deficiency presenting with hemolysis, nephritis, and asplenia. J Pediatr Hematol Oncol.

[15] Yachie A (1999). Oxidative stress causes enhanced endothelial cell injury in human heme oxygenase-1 deficiency. J Clin Invest.

[16] Dixon SJ (2012). Ferroptosis: an iron-dependent form of nonapoptotic cell death. Cell.

[17] Escuder-Rodriguez JJ (2024). Ferroptosis: biology and role in gastrointestinal disease. Gastroenterology.

[18] Stockwell BR (2022). Ferroptosis turns 10: emerging mechanisms, physiological functions, and therapeutic applications. Cell.

[19] Zhang R (2023). Ferroptosis in gastrointestinal cancer: from mechanisms to implications. Cancer Lett.

[20] Schwartz AJ (2021). Hepcidin sequesters iron to sustain nucleotide metabolism and mitochondrial function in colorectal cancer epithelial cells. Nat Metab.

[21] Liu Z (2023). Iron promotes glycolysis to drive colon tumorigenesis. Biochim Biophys Acta Mol Basis Dis.

[22] Vallelian F (2015). Proteasome inhibition and oxidative reactions disrupt cellular homeostasis during heme stress. Cell Death Differ.

[23] Dieleman LA (1998). Chronic experimental colitis induced by dextran sulphate sodium (DSS) is characterized by Th1 and Th2 cytokines. Clin Exp Immunol.

[24] Sheikh SZ (2011). An anti-inflammatory role for carbon monoxide and heme oxygenase-1 in chronic Th2-mediated murine colitis. J Immunol.

[25] Hegazi RA (2005). Carbon monoxide ameliorates chronic murine colitis through a heme oxygenase 1-dependent pathway. J Exp Med.

[26] Onyiah JC (2013). Carbon monoxide and heme oxygenase-1 prevent intestinal inflammation in mice by promoting bacterial clearance. Gastroenterology.

[27] Kanehisa M, Goto S (2000). KEGG: Kyoto encyclopedia of genes and genomes. Nucleic Acids Res.

[28] Sun Y (2018). Glutathione depletion induces ferroptosis, autophagy, and premature cell senescence in retinal pigment epithelial cells. Cell Death Dis.

[29] Hedblom A (2019). Heme detoxification by heme oxygenase-1 reinstates proliferative and immune balances upon genotoxic tissue injury. Cell Death Dis.

[30] Planell N (2013). Transcriptional analysis of the intestinal mucosa of patients with ulcerative colitis in remission reveals lasting epithelial cell alterations. Gut.

[31] Barton SG (2003). Expression of heat shock protein 32 (hemoxygenase-1) in the normal and inflamed human stomach and colon: an immunohistochemical study. Cell Stress Chaperones.

[32] Paul G (2005). Analysis of intestinal haem-oxygenase-1 (HO-1) in clinical and experimental colitis. Clin Exp Immunol.

[33] Takagi T (2008). Increased intestinal expression of heme oxygenase-1 and its localization in patients with ulcerative colitis. J Gastroenterol Hepatol.

[34] Barrera G (2012). Oxidative stress and lipid peroxidation products in cancer progression and therapy. ISRN Oncol.

[35] Hayes JD (2020). Oxidative stress in cancer. Cancer Cell.

[36] Singhal R (2021). HIF-2α activation potentiates oxidative cell death in colorectal cancers by increasing cellular iron. J Clin Invest.

[37] Tanaka T (2003). A novel inflammation-related mouse colon carcinogenesis model induced by azoxymethane and dextran sodium sulfate. Cancer Sci.

[38] Wei R (2021). Tagitinin C induces ferroptosis through PERK-Nrf2-HO-1 signaling pathway in colorectal cancer cells. Int J Biol Sci.

[39] Kumar S, Bandyopadhyay U (2005). Free heme toxicity and its detoxification systems in human. Toxicol Lett.

[40] Sobin LH (1985). The histopathology of bleeding from polyps and carcinomas of the large intestine. Cancer.

[41] Vega PN (2022). Cancer-associated fibroblasts and squamous epithelial cells constitute a unique microenvironment in a mouse model of inflammation-induced colon cancer. Front Oncol.

[42] Sirvinskas D (2022). Single-cell atlas of the aging mouse colon. iScience.

[43] Haber AL (2017). A single-cell survey of the small intestinal epithelium. Nature.

[44] Schmitt TH (1993). Hemin-induced lipid membrane disorder and increased permeability: a molecular model for the mechanism of cell lysis. Arch Biochem Biophys.

[45] Adedoyin O (2018). Heme oxygenase-1 mitigates ferroptosis in renal proximal tubule cells. Am J Physiol Renal Physiol.

[46] Kwon MY (2015). Heme oxygenase-1 accelerates erastin-induced ferroptotic cell death. Oncotarget.

[47] Nishizawa H (2020). Ferroptosis is controlled by the coordinated transcriptional regulation of glutathione and labile iron metabolism by the transcription factor BACH1. J Biol Chem.

[48] Magtanong L (2022). Context-dependent regulation of ferroptosis sensitivity. Cell Chem Biol.

[49] Dang D (2022). Heme induces intestinal epithelial cell ferroptosis via mitochondrial dysfunction in transfusion-associated necrotizing enterocolitis. FASEB J.

[50] Han S (2022). HO-1 contributes to luteolin-triggered ferroptosis in clear cell renal cell carcinoma via increasing the labile iron pool and promoting lipid peroxidation. Oxid Med Cell Longev.

[51] Yang J (2021). Cetuximab promotes RSL3-induced ferroptosis by suppressing the Nrf2/HO-1 signalling pathway in KRAS mutant colorectal cancer. Cell Death Dis.

[52] Suliman HB (2017). Mitochondrial quality-control dysregulation in conditional HO-1^-/-^ mice. JCI Insight.

[53] Madison BB (2002). Cis elements of the villin gene control expression in restricted domains of the vertical (crypt) and horizontal (duodenum, cecum) axes of the intestine. J Biol Chem.

[54] Schneider CA (2012). NIH image to ImageJ: 25 years of image analysis. Nat Methods.

[55] Schaefer REM (2022). Disruption of monocyte-macrophage differentiation and trafficking by a heme analog during active inflammation. Mucosal Immunol.

[56] Hall CHT (2020). Creatine transporter, reduced in colon tissues from patients with inflammatory bowel diseases, regulates energy balance in intestinal epithelial cells, epithelial integrity, and barrier function. Gastroenterology.

[57] Van den Berg JW (1988). Automating the quantification of heme in feces. Clin Chem.

[58] Barrett T (2013). NCBI GEO: archive for functional genomics data sets--update. Nucleic Acids Res.

[59] https://www.R-project.org/.

[60] Hao Y (2021). Integrated analysis of multimodal single-cell data. Cell.

[61] McGinnis CS (2019). DoubletFinder: doublet detection in single-cell RNA sequencing data using artificial nearest neighbors. Cell Syst.

[63] Gu Z (2016). Complex heatmaps reveal patterns and correlations in multidimensional genomic data. Bioinformatics.

[64] Finak G (2015). MAST: a flexible statistical framework for assessing transcriptional changes and characterizing heterogeneity in single-cell RNA sequencing data. Genome Biol.

[65] Durinck S (2009). Mapping identifiers for the integration of genomic datasets with the R/Bioconductor package biomaRt. Nat Protoc.

