# Supplementary figures and images for: Epithelial HO-1 regulates iron availability and promotes colonic tumorigenesis in a context-dependent manner
Source: JCI Insight. 2025 Dec 17;11(3):e181032. doi: 10.1172/jci.insight.181032 (PMC12892898; doi:10.1172/jci.insight.181032)

# Full unedited gel for Figure 2

4-HNE  
(Abcam)

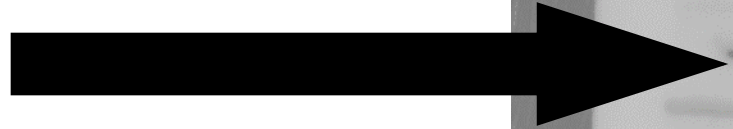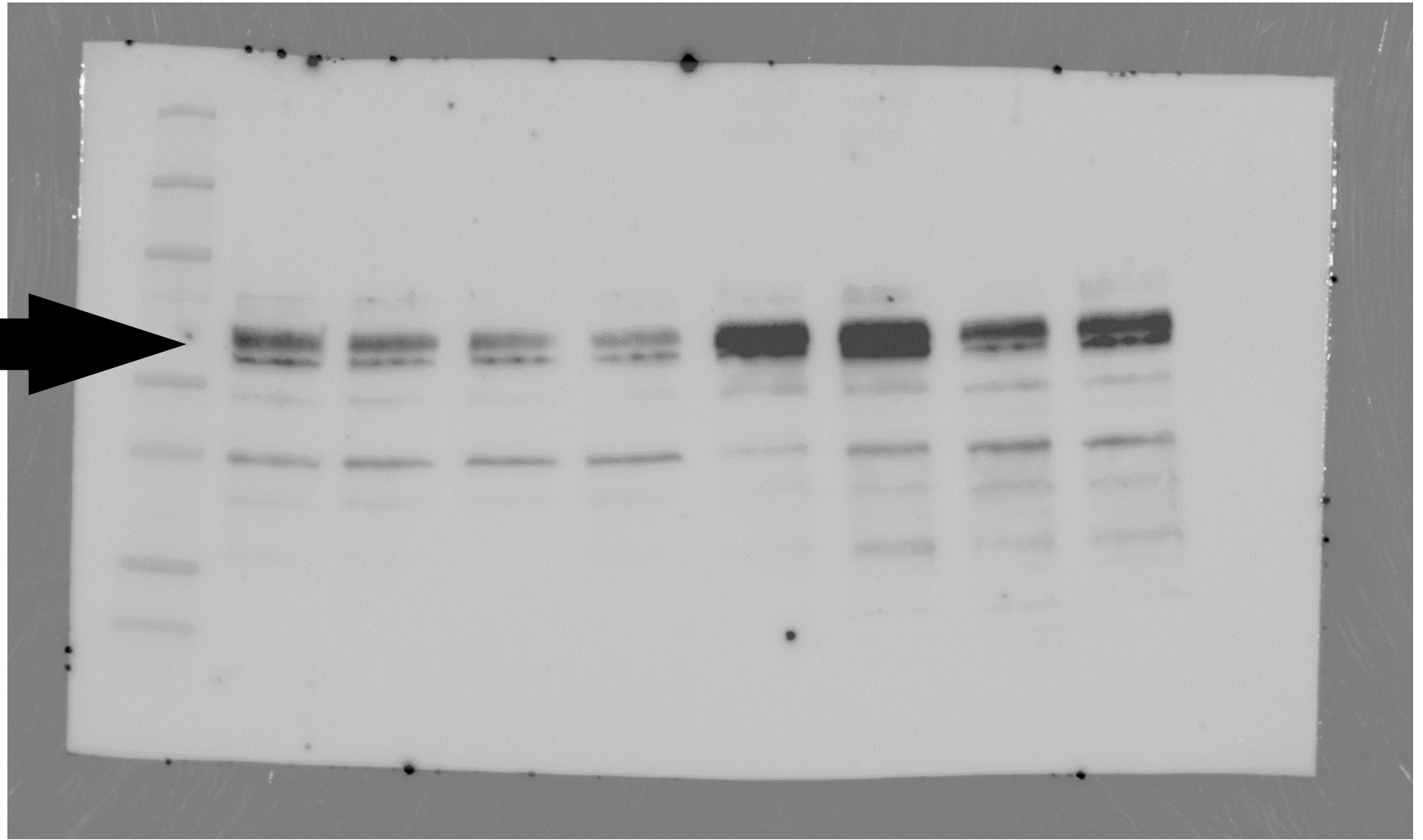

# Full unedited gel for Figure 2

$\beta$ -tubulin  
(Sigma-Aldrich)

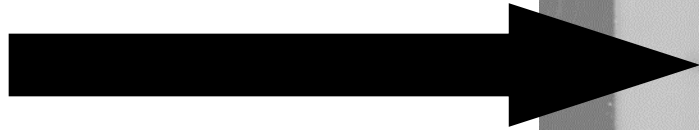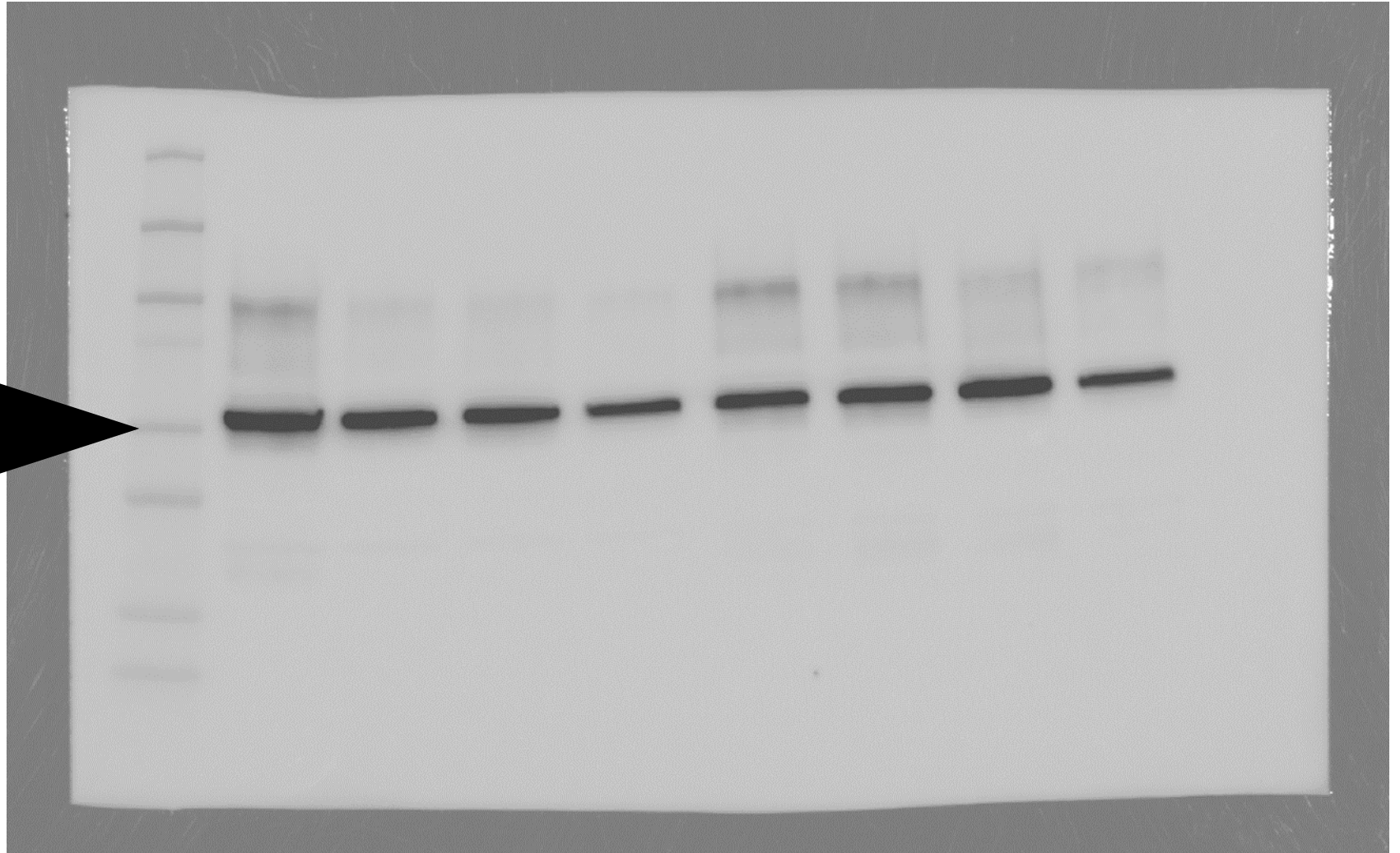

Supplement: Unedited blot and gel images [file jciinsight-11-181032-s192.pdf]
